# Morphological and mechanical properties of the human triceps surae aponeuroses taken from elderly cadavers: Implications for muscle-tendon interactions

**DOI:** 10.1371/journal.pone.0211485

**Published:** 2019-02-08

**Authors:** Xiyao Shan, Shun Otsuka, Tomiko Yakura, Munekazu Naito, Takashi Nakano, Yasuo Kawakami

**Affiliations:** 1 Faculty of Sport Sciences, Waseda University, Tokorozawa, Saitama, Japan; 2 Department of Anatomy, Aichi Medical University, Nagakute, Aichi, Japan; University of Pittsburgh, UNITED STATES

## Abstract

The human triceps surae (two gastrocnemii and soleus) has aponeuroses in the proximal and distal aspects, the latter of which insert into the calcaneus by sharing the common Achilles tendon. These tendinous tissues are known to have elasticity and upon muscle contraction the aponeurosis is stretched both longitudinally (along the muscle’s line of action) and transversely. Higher aponeurosis transverse deformability has been documented, but there is a paucity of information on the morphology and mechanical properties of human aponeurosis. This study aimed to identify morphological and mechanical characteristics of the human triceps surae aponeuroses. Twenty-five triceps surae muscle-tendon units were procured from 13 human donors (formalin fixed, 6 males, 7 females) aged 67–91 years. Specimens of aponeuroses were excised from the eight regions (posterior and anterior regions of the gastrocnemius medialis and lateralis, medial and lateral parts of soleus; proximal, middle, and distal sites each, 2–4 cm × 2–4 cm). Aponeurosis thickness was measured using a digital caliper. Uniaxial tensile tests were implemented to determine the mechanical properties of specimens loaded longitudinally (along the muscle’s line of action) and transversely. The aponeurosis thickness showed significant differences between muscles and sites, while Young’s modulus showed direction-dependent (longitudinal vs. transverse) differences within sites. Results show different morphology and mechanical properties of aponeuroses between synergist muscles. The reason for site-dependent differences in stiffness is due to a reduced aponeurosis thickness rather than a reduction in the material property. The anisotropic elastic feature (differences between longitudinal and transverse directions) of the aponeuroses was more pronounced than previous *in vivo* findings, suggesting inherent material design of the aponeurosis that matches three-dimensional contractile behavior of muscle fibers.

## Introduction

The tendinous tissues (tendon and aponeuroses) play a significant role in human movements functioning as a spring and contributing to energy saving and power enhancement of the muscle-tendon unit [1–3]. Unlike the cord-like structure of tendon, the sheet-like structure of aponeurosis serves as an attachment site of muscle fascicles on the surface of a muscle belly, and can bear the pressure and tension during muscle contraction [4, 5]. During muscle contraction, the aponeurosis is stretched both in the longitudinal (along the muscle’s line of action) and the transverse directions, and higher transverse deformability has been documented in previous studies [6–8]. However, the intrinsic bidirectional differences in the human aponeurosis mechanical properties remain unclear. The triceps surae (two gastrocnemii and soleus) has aponeuroses in the proximal and distal aspects, the latter of which ultimately insert into the calcaneus by sharing the common Achilles tendon. Gastrocnemii have been considered at higher risk for strains than soleus because of different architecture between gastrocnemii (biarticular) and soleus (monoarticular) muscles [9]. Furthermore, it is necessary to delineate the differences in aponeurotic mechanics between gastrocnemii and soleus. Previous studies have reported anisotropic (different material properties in the transverse and longitudinal directions) and inhomogeneous properties of the triceps surae aponeuroses [10, 11], and site-dependent differences of aponeuroses strains have been found during human movements through *in vivo* studies [12–14]. Few studies, however, focused on the site-dependent morphology of triceps surae aponeuroses. While a cadaveric study revealed the geometry and thickness distribution of the triceps surae aponeuroses using one leg of an elderly male cadaver [15], there is still a paucity of information on the intrinsic morphological and mechanical properties of human aponeurosis. Another previous study showed the gradient in aponeurosis thickness appeared to match the gradient in tension transmitted along aponeurosis structure [4], therefore we hypothesized that thinner aponeuroses are more compliant than thicker aponeuroses. Additionally, it remains unknown whether or not the anisotropy, inhomogeneity and inter-muscular differences of human aponeuroses are due to differences of their morphological and mechanical properties. These issues cannot be approached by *in vivo* studies where one cannot directly and accurately measure intrinsic morphological and mechanical properties of aponeuroses.

A tensile test is an important measure to evaluate the mechanical properties of the soft tissues, and has been applied to obtain the stiffness and/or Young’s modulus of anisotropic tissues along different directions [16–18]. As a viscoelastic tissue, not only stiffness and Young's modulus, but hysteresis also needs to be considered to delineate the mechanical properties of aponeuroses. However, the tensile properties of human triceps surae aponeuroses directly measured *ex-situ* have not been reported. This study therefore aimed to identify the morphological and mechanical characteristics of the human triceps surae aponeuroses, in particular, the site- and direction-dependent differences and differences between gastrocnemii and soleus.

## Materials and methods

### Materials

**Fig 1:**
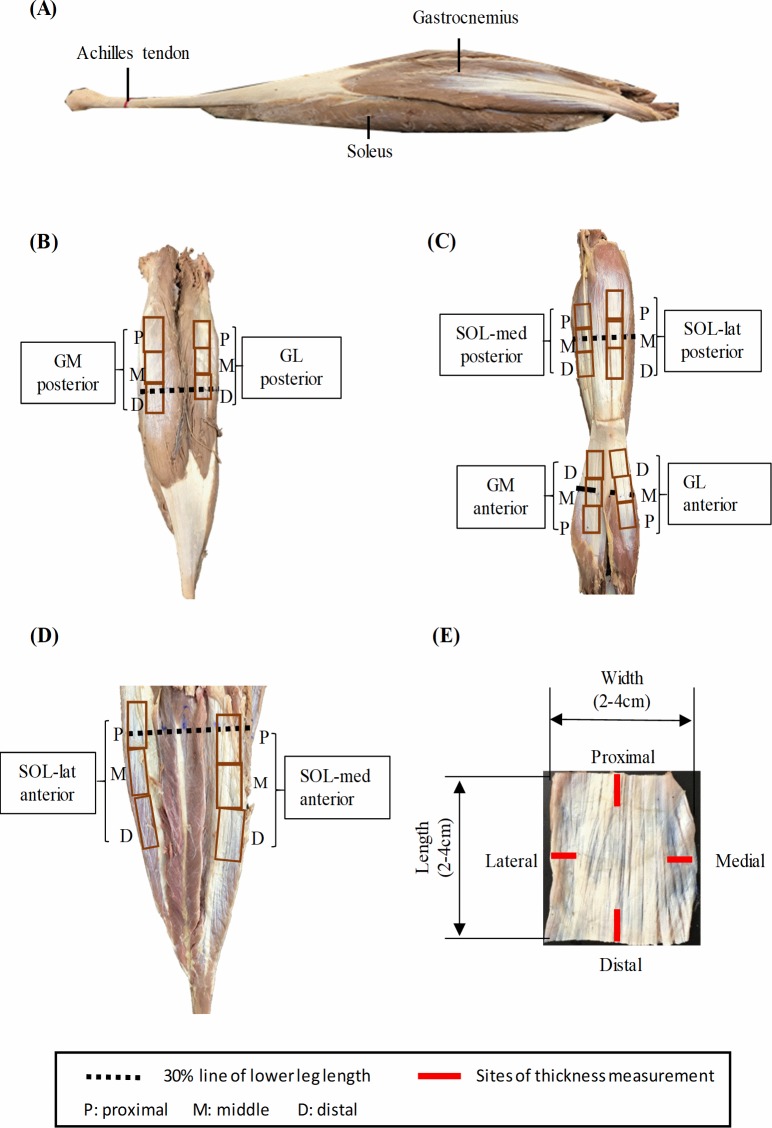
Specimen preparation, three size-matched rectangular specimens of aponeuroses were dissected from three sites of each region. (A) Lateral sagittal view of triceps surae muscle-tendon unit with aponeuroses. (B) Posterior view of posterior part of gastrocnemii. (C) Posterior view of anterior part of gastrocnemii and posterior part of soleus. (D) Anterior view of anterior part of soleus. (E) One typical specimen of aponeurosis. GM gastrocnemius medialis, GL gastrocnemius lateralis, SOL-med medial part of soleus, SOL-lat lateral part of soleus.

This study was approved (2017-M001) by the ethics committee of Aichi Medical University. Twenty-five triceps surae muscle-tendon units (324.2 ± 98.8 g, mean ± standard deviation) were procured from 13 human donors (formalin fixed, 6 males, 7 females) aged 67–92 years (82.2 ± 10.1, mean ± standard deviation). The sample size (n = 25) exceeded the necessary number of samples for this study (n = 24, determined by power analysis with the power: 0.80 and effect size *f* = 0.75) [19]. The cadavers were donated to Aichi Medical University, Aichi, Japan. Before they died, the donors signed the informed consent agreeing to body donation and its use for research. The cadavers were embalmed by using 20% formaldehyde, a Porti-boy pump with cannula was used to inject embalming fluid into the body through the femoral artery (toward the feet) and common carotid artery (toward the cephalad). Once embalming was completed, the body was placed in a sealed plastic body bag and stored at room temperature [20]. Specimens of aponeuroses were excised from eight regions: posterior and anterior regions of gastrocnemius medialis (GM), gastrocnemius lateralis (GL), medial part of soleus (SOL-med), lateral part of soleus (SOL-lat). In each region, three size-matched rectangular pieces of aponeuroses (2–4 cm x 2–4 cm) were harvested from the proximal, middle and distal sites (Fig 1B, 1C and 1D). The muscles and aponeuroses specimens were kept moist by using Alcohol (50% by volume) throughout the dissection process [18].

### Measurements

**Fig 2:**
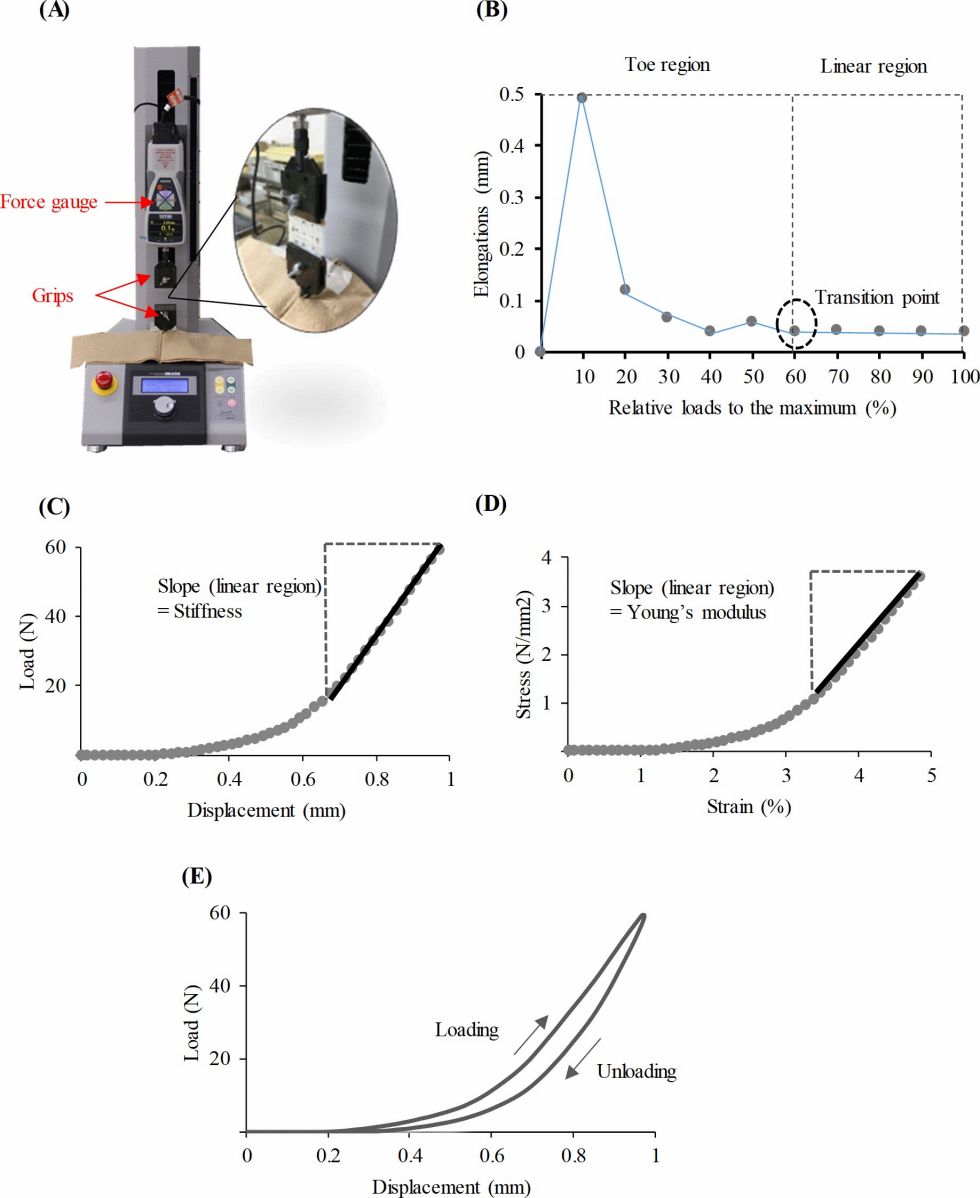
Mechanical testing and data process. (A) A tensile test machine with an inset showing a gripped specimen. (B) A relationship curve between relative loads (to maximal) and elongations. (C) A representative load-displacement curve recorded by the tensile test. (D) A representative stress-strain curve recorded by the tensile test. (E) A representative load-displacement curve during tensile test. The arrows indicate loading and unloading directions.

The average thickness of each aponeurosis specimen was calculated after measuring the thickness at four different sites (Fig 1E) of each specimen by using a digital vernier caliper (LIXIL VIVA, Japan) [21, 22]. It was not possible to perfectly remove muscle fibers from each sample (otherwise we could break it) but we took great care to trim them as much as we could while not destroying the aponeurosis, and to avoid inclusion of remaining fibers during thickness measurement. For each site, we repeated at least 3 times to avoid variations between trials, then the value of thickness was recorded. The specimen’s average thickness and width (or length) were used to calculate the cross-sectional area of the specimen. The uniaxial tensile test was implemented by using an instrument (IMADA CO., LTD, Japan) that was equipped with one test stand and two sets of force gauges (1: ZTA-500N, 1000Hz; 2: ZTA-5N, 1000Hz, Fig 2A). Before placing and fixing each specimen of aponeurosis on the instrument, sandpapers (5 cm × 0.5 cm) were glued to the top and bottom ends of the specimen to prevent slipping from the thin film grips during the tensile test [23] and the synchronous loading-unloading curve that was displayed real-time during measurement to ensure slippage did not occur. In such a case with an irregular shape of the curve, the test was repeated after adjusting the grip interface. The specimens of aponeuroses were loaded longitudinally (along the muscle’s line of action) and transversely, to avoid the order effect of testing in one to the other directions, the order of testing on the two directions was randomized and counterbalanced [18]. According to the previous study [16] and our pilot experiment, the aponeuroses were expected to be stiffer in the longitudinal than the transverse direction. Thus, the force gauge 1 (ZTA-500N) and 2 (ZTA-5N) were used for the longitudinal and transverse direction test respectively. The load and displacement during the tensile test were displayed simultaneously in the kit software. The speed was set at 25 mm·min^-1^ [18] both for the stretch and relaxation, and five identical cycles were performed during the cyclic tensile test. All the tensile tests were carried out at a room temperature (20–26°C).

### Analyses

The data of all tested specimens were used for the analyses unless the specimens were damaged during dissection or tensile test processes. Out of the five cycles of the tensile test, forces obtained from the first and second cycles were slightly smaller due to the initial aponeuroses settling within the film grips of the force measurement instrument. We used only the third cycle for analysis in order not to introduce possible force reduction in the last two cycles (4th and 5th) [16]. The stress (σ) and strain (ε) were calculated using the following equations.

σ=F/A (1)

ε=(l−L)/L (2)

Where *F* is the tensile force, *A* is the initial (unloaded) cross-sectional area of each specimen, *l* and *L* are the final and initial lengths of the aponeurosis, respectively. The initial length (resting length) of each specimen was determined using the same threshold force (when the load cell reached 0.1N).

For the load-displacement and stress-strain curves, the linear region was identified based on a previous study [24] as follows: First, loads were normalized to the maximum load and then elongation was recorded at relative loads of 10%, 20%, 30%, 40%, 50%, 60%, 70%, 80%, 90%, and 100%. Secondly, from the relationship between the relative loads and elongations, slopes at adjacent data points were calculated to find the transition point (when the slope is zero) from the toe- to linear-region (Fig 2B). The stiffness (N/mm) and tensile modulus (MPa) were calculated from the slope of the linear region of the load-displacement relationship curve and stress-strain relationship curve respectively (Fig 2C and 2D), and were used as mechanical variables of the aponeuroses. The mechanical hysteresis was calculated in the same way as in a previous study [25] using Eq (3).

$$Hysteresis\;(\%)=[(S_{loading}-S_{unloading})/S_{loading}]\times 100$$ (3)

Where *S*_*loading*_ is the area under the loading curve and *S*_*unloading*_ is the area under the unloading curve (Fig 2E). All calculations were performed by using the software Origin 9.0 (OriginLab, Northampton, MA, USA).

We did not find significant sex differences in any of the measurements and the derived parameters, therefore we pooled the data for males and females.

### Statistics

All the data are shown as mean ± standard deviation. Accounting for unequal sample size (number of specimens) between sites, and variations among and within individuals, a one-way factorial mixed-model ANOVA (analysis of variance) with individual as a random effect and sites (proximal, middle and distal) as fixed factors was used to determine the site-dependent differences in thickness. To test the site- and direction-dependent differences in stiffness, Young’s modulus and hysteresis, a two-way factorial mixed-model ANOVA with individual as a random effect and fixed factors [sites (proximal, middle, distal) and directions (longitudinal and transverse)] was performed. To compare across regions (posterior and anterior) within a muscle and then compare the differences among muscles (GM, GL, SOL-med and SOL-lat), the average [proximal/middle/distal (P/M/D)] thickness and Young’s modulus were calculated, and a two-way factorial mixed-model ANOVA with individual as a random effect and fixed factors [muscles (GM, GL, SOL-med and SOL-lat) and regions (posterior and anterior)] was used. A post-hoc test (Bonferroni) was performed when appropriate. All the statistical analyses were performed using SPSS Statistics 24.0 (IBM SPSS Statistics, SPSS Inc., Chicago, USA). The significance level was set at *α* < 0.05.

## Results

### Thickness

**Fig 3:**
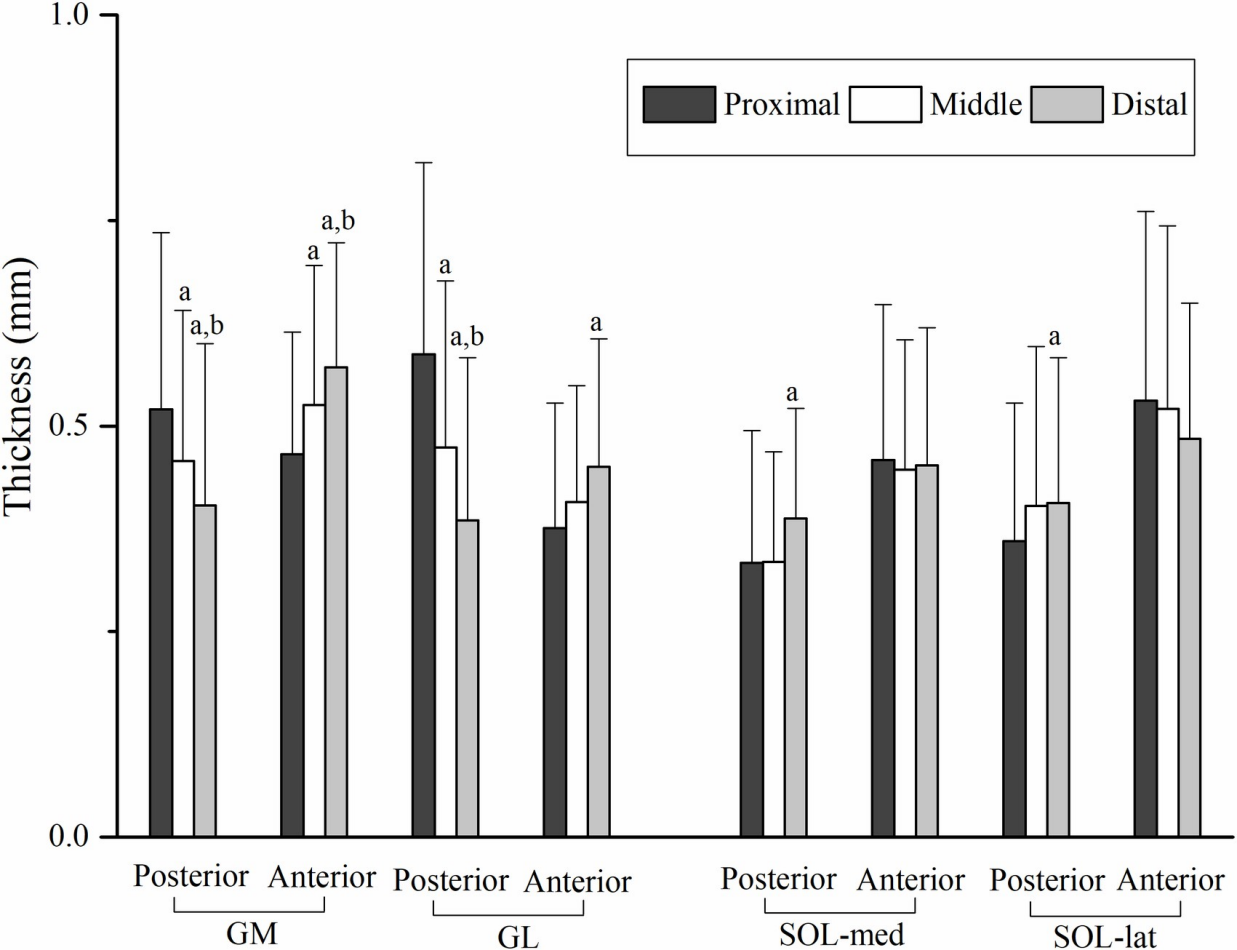
Average (mean + s.d.) thickness of aponeuroses from proximal to distal sites in each region. a: denotes different from proximal site, *p* < 0.05; b: denotes different from middle site, *p* < 0.05. GM gastrocnemius medialis, GL gastrocnemius lateralis, SOL-med medial part of soleus, SOL-lat lateral part of soleus.

In the posterior and anterior regions of GM and GL, significant differences among the proximal (P), middle (M) and distal (D) sites were showed [posterior region of GM: P (0.52 ± 0.21 mm) > M (0.46 ± 0.18 mm) > D (0.40 ± 0.20 mm), posterior region of GL: P (0.59 ± 0.23 mm) > M (0.47 ± 0.20 mm) > D (0.39 ± 0.20 mm), anterior region of GM: D (0.57 ± 0.15 mm) > M (0.53 ± 0.17 mm) > P (0.47 ± 0.15 mm), and anterior region of GL: D (0.45 ± 0.16 mm) > P (0.38 ± 0.15 mm), *p* < 0.01]. In the posterior regions of SOL-med and SOL-lat, the proximal site was significantly thinner than the distal site [SOL-med: D (0.39 ± 0.13 mm) > P (0.33 ± 0.16 mm), SOL-lat: D (0.41 ± 0.18 mm) > P (0.36 ± 0.17 mm), *p* < 0.05], and there was no significant difference among sites in the anterior regions of SOL-med and SOL-lat (Fig 3).

**Fig 4:**
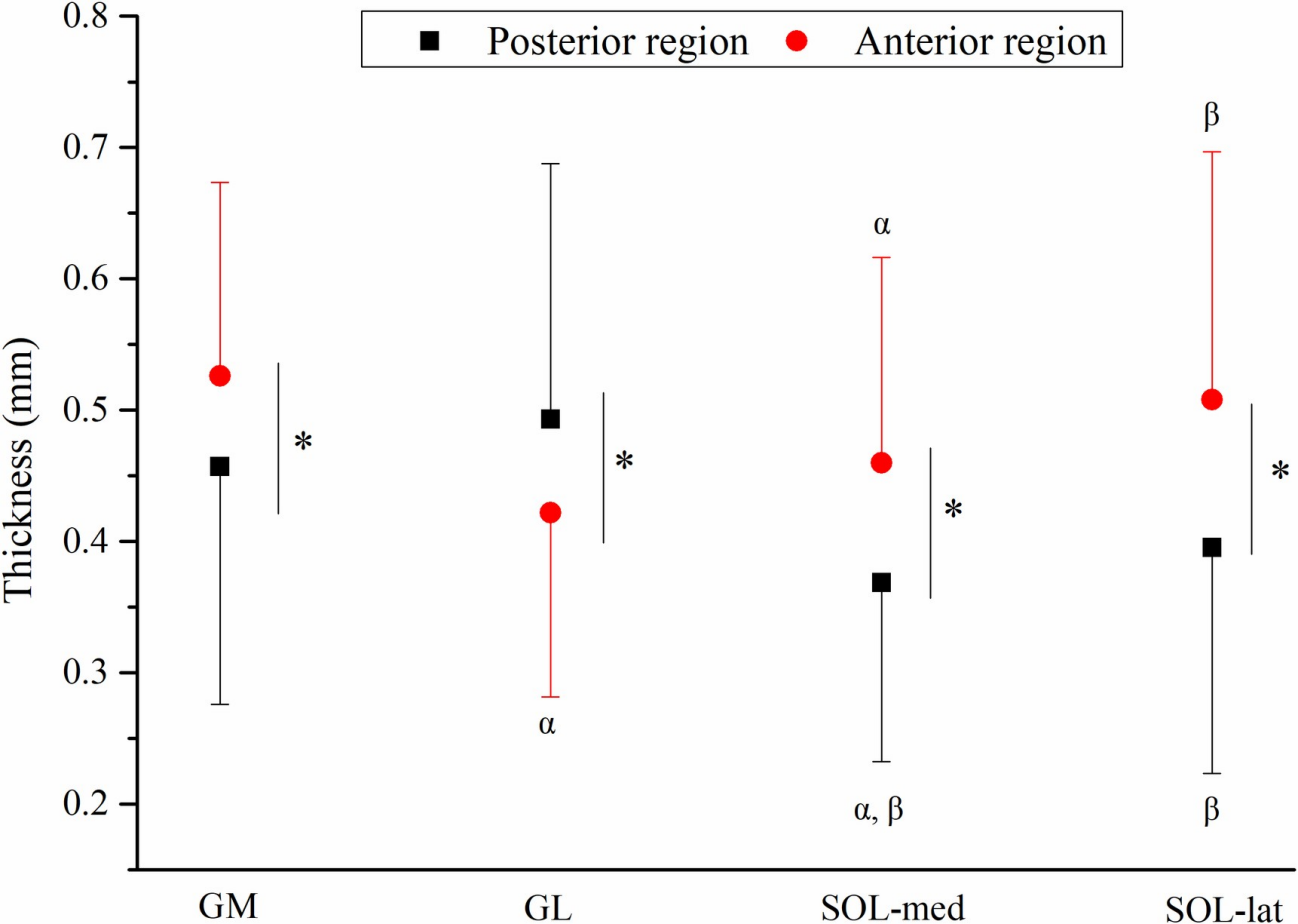
Average (proximal/middle/distal sites, mean ± s.d.) thickness of aponeuroses in posterior and anterior regions of the triceps surae. *: denotes differences between posterior and anterior regions, *p* < 0.05; α: denotes different from GM, *p* < 0.05; β: denotes different from GL, *p* < 0.05. GM gastrocnemius medialis, GL gastrocnemius lateralis, SOL-med medial part of soleus, SOL-lat lateral part of soleus.

For the average (across the P/M/D sites) thickness of aponeuroses in the posterior and anterior regions of the triceps surae (GM, GL, SOL-med and SOL-lat), there was significant muscle × region interaction (*p*<0.001) while the main effects of muscle (*p*<0.001) and region (*p*<0.001) were both significant (Fig 4). In the posterior regions, GL = GM > SOL-med = SOL-lat (*p*<0.01, Fig 4), and in the anterior regions, GM = SOL-lat > GL = SOL-med (*p* < 0.05, Fig 4).

### Stiffness

Stiffness in all regions of the triceps surae aponeuroses showed significant differences between the longitudinal and transverse directions (*p* < 0.001, Table 1). Significant differences in the longitudinal stiffness among the proximal, middle and distal sites were showed in the posterior (P = M > D, *p* < 0.01) and anterior (P = M > D, *p* < 0.01) regions of GM and anterior region (P < M = D, *p* < 0.05) of GL (Table 1).

**Table 1:** Mean and standard deviations of the stiffness values for the tested specimens (N/mm).

	GM-posterior		GM-anterior		SOL-med-posterior		SOL-med-anterior	
	L	T	L	T	L	T	L	T
Proximal	114.2±36.7	0.7±1.4^#^	107.1±33.3	0.9±0.9^#^	83.7±27.3	0.9±0.7^#^	74±26.6	0.9±1.5^#^
Middle	114.3±37.5	0.4±0.7^#^	125.4±40.1	0.6±0.5^#^	89.6±38	0.9±1.1^#^	78.7±32.3	0.7±0.6^#^
Distal	83.3±34.8^a^^,^^b^	0.3±0.4^#^	143.6±45.2^a^	0.8±1.1^#^	106.9±35.9	0.8±0.7^#^	69±26.5	1±0.9^#^
	GL-posterior		GL-anterior		SOL-lat-posterior		SOL-lat-anterior	
	L	T	L	T	L	T	L	T
Proximal	108.2±35	0.7±0.6^#^	102±45.9	0.8±0.9^#^	64.5±29.1	1.2±1.3^#^	75.9±27.2	0.8±0.9^#^
Middle	109.7±36.1	0.9±1^#^	144.7±41.4^a^	1.2±1.3^#^	80.9±35.1	1.5±1.4^#^	73.9±30.2	0.8±0.7^#^
Distal	87.5±18.7	0.8±0.5^#^	129.6±46.7^a^	1.8±1.6^#^	88.4±34.2	1.3±1.1^#^	80.1±31.3	1.5±1.2^#^

#: denotes different from longitudinal direction, p < 0.05

a: differences from proximal site

b: differences from middle site.

GM gastrocnemius medialis, GL gastrocnemius lateralis, SOL-med medial part of soleus, SOL-lat lateral part of soleus, L longitudinal, T transverse.

### Young’s modulus

For the Young's modulus, there was no significant interaction between sites and directions in any regions of triceps surae aponeuroses, while the values in the longitudinal direction were significantly higher than those in the transverse direction in all regions (*p* < 0.001, Table 2).

**Table 2:** Mean and standard deviations of the Young's modulus values for the tested specimens (MPa).

	GM-posterior		GM-anterior		SOL-med-posterior		SOL-med-anterior	
	L	T	L	T	L	T	L	T
Proximal	207.1±118.7	0.6±0.9^#^	198.2±118.3	1.1±1.2^#^	264.3±155.5	1.2±1^#^	211.6±166.1	0.7±0.9^#^
Middle	210.4±96	0.4±0.6^#^	196.1±89.2	0.8±0.9^#^	261.7±196.9	1.4±1.6^#^	199.1±113.1	0.8±0.9^#^
Distal	182.8±106.6	0.6±0.6^#^	207.5±103.2	0.5±0.5^#^	262±127.7	0.9±0.9^#^	177.6±120	0.8±0.5^#^
	GL-posterior		GL-anterior		SOL-lat-posterior		SOL-lat-anterior	
	L	T	L	T	L	T	L	T
Proximal	289.1±214.5	0.5±0.5^#^	283.9±168.9	1.1±1.1^#^	185.7±132.2	2.3±2.2^#^	164.1±124.6	0.8±0.9^#^
Middle	280±104.4	1±1.2^#^	323.3±151	1.4±1.4^#^	225.4±168.8	2±1.9^#^	197.9±181.2	0.7±0.5^#^
Distal	191.9±66.5	1.3±0.7^#^	304.6±157.1	1.9±1.9^#^	256.2±159.2	1.9±1.9^#^	173.8±75.6	1.3±1.4^#^

#: denotes different from longitudinal direction, p < 0.05; GM gastrocnemius medialis, GL gastrocnemius lateralis, SOL-med medial part of soleus, SOL-lat lateral part of soleus, L longitudinal, T transverse.

**Fig 5:**
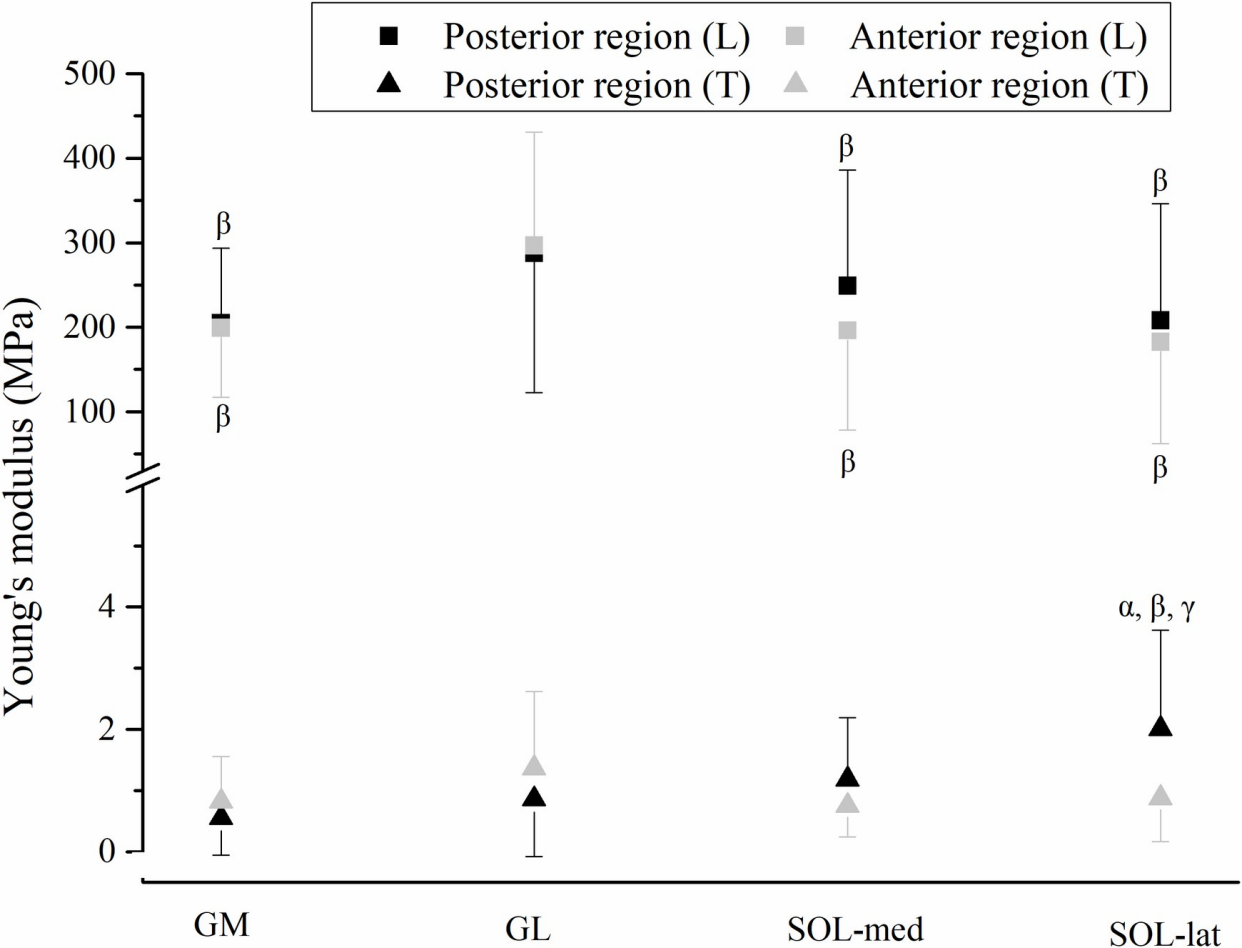
Average (proximal/middle/distal sites, mean ± s.d.) Young’s modulus in L (longitudinal) and T (transverse) directions of aponeuroses in posterior and anterior regions of the triceps surae. α: denotes different from GM, *p* < 0.05; β: denotes different from GL, *p* < 0.05; γ: denotes different from SOL-med, *p* < 0.05. GM gastrocnemius medialis, GL gastrocnemius lateralis, SOL-med medial part of soleus, SOL-lat lateral part of soleus.

For the average (across the P/M/D sites) Young’s modulus of aponeuroses in posterior and anterior regions of the triceps surae, in the longitudinal direction, the muscle × region interaction showed no significance while the main effect of muscle was significant (posterior region: GL > GM = SOL-med = SOL-lat, and anterior region: GL > GM = SOL-med = SOL-lat, *p* < 0.001, Fig 5). In the transverse direction, the Young’s modulus of posterior region of SOL-lat was significantly higher than GM (*p* < 0.001), GL (*p* = 0.003), and SOL-med (*p* = 0.012) (Fig 5).

### Hysteresis

The hysteresis in the transverse direction was significantly larger than in the longitudinal direction at the middle and distal sites of posterior region of GM (*p <* 0.001), proximal and middle sites of anterior region of GL (*p* = 0.001), all three sites of posterior region of GL (*p* = 0.01) and SOL-med (*p* = 0.001), anterior region of SOL-med (*p* = 0.002) and SOL-lat (*p* = 0.009). In contrast, the hysteresis in the distal site of anterior region of GL was higher in the longitudinal than the transverse direction (*p* = 0.005, Table 3).

**Table 3:** Mean and standard deviations of the hysteresis values for the tested specimens (%).

	GM-posterior		GM-anterior		SOL-med-posterior		SOL-med-anterior	
	L	T	L	T	L	T	L	T
Proximal	41.1±8.5	47.5±20.7	38±8.4	40.5±16.8	26.1±7.8	40.6±20.3^#^	31.3±7.1	45.2±15.1^#^
Middle	33.9±9.9	54±17.8^#^	45.2±8.8	40.2±9.6	33.7±11.5	38.7±18.7^#^	36.8±11.7	40.4±12.2^#^
Distal	28.2±8.7	53.8±15.8^#^	46.6±7^a^	48.6±18.3^a^	34.8±8.2	38.8±12.9^#^	33.7±8.7	44.5±21.6^#^
	GL-posterior		GL-anterior		SOL-lat-posterior		SOL-lat-anterior	
	L	T	L	T	L	T	L	T
Proximal	41.4±6.9	46.6±19.2^#^	29±8	50.3±21.3^#^	29.5±7.9	39.7±17.8	37.9±9.6	44.7±16.4^#^
Middle	36.5±8.2	37.8±16.4^#^	34.5±8.7	39.6±18.2^#^	35.3±9.9	32.4±9.7	38.1±11.3	48.5±16.4^#^
Distal	29±4.9^a^	41±9.4^a^^,^ ^#^	40.7±8.7	35.8±12.4^#^	35.6±10.2	39.1±17.4	34.6±10.3^b^	37.9±12^b^^,^ ^#^

#: denotes different from longitudinal direction, p < 0.05

a: differences from proximal site

b: differences from middle site.

GM gastrocnemius medialis, GL gastrocnemius lateralis, SOL-med medial part of soleus, SOL-lat lateral part of soleus, L longitudinal, T transverse.

## Discussion

In the present study, we measured morphological and mechanical properties of the human triceps surae aponeuroses by using uniaxial tensile tests, and found the site- and direction-dependent differences in anisotropy and heterogeneity of aponeurotic tissues. In addition, differences of material properties of aponeuroses in synergist muscles (gastrocnemii and soleus) were provided which would help us better understand the contributing factors for the force transmission in muscle-aponeurosis-tendon complex.

### Site-dependent differences of morphological and mechanical properties of aponeurosis

**Fig 6:**
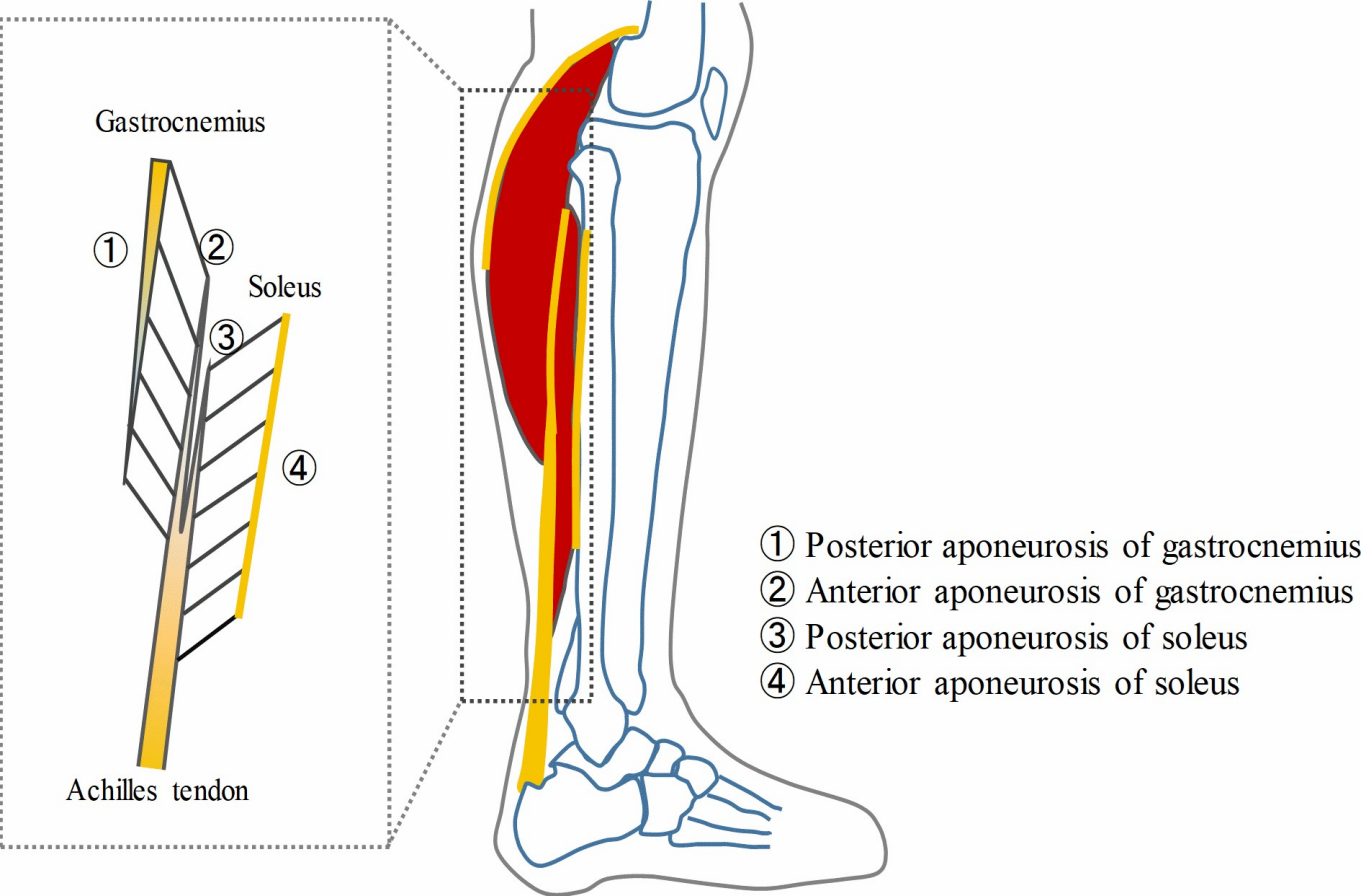
Sagittal view of the triceps surae and schematic representation of aponeurosis thickness distribution. Orange color (with gradation) denotes tendinous tissue (i.e. tendon or aponeurosis).

In each region of the triceps surae aponeuroses, except for the anterior regions of soleus, the thickness distributed inhomogeneously from the proximal to distal sites (Figs 3 and 6), which is consistent with the previous study [15] that showed site differences of soleus aponeuroses thickness. Being continuous with the free portion of the tendon and further extending upon the muscle belly, the aponeurosis not only acts to transmit forces in its longitudinal direction, but also bears all the possible tension and deformation of the contracting muscle belly in the longitudinal as well as transverse direction [26]. A flat sheet-like structure with anisotropic stiffness covering the muscle belly surface would contribute to such contraction-induced muscle behavior.

As for the longitudinal direction of the aponeurosis, the aponeurotic parts around the proximal and distal ends of muscle belly would experience forces developed from the majority of muscle fibers, while the parts near the termination of the aponeurosis experience forces with far fewer fibers [27]. During muscle contraction, the aponeurosis would be stretched more around the ends of muscle belly while less around the termination of the aponeurosis. If however the aponeurosis exhibits a similar stretch regardless of its part, it should have higher stiffness around the muscle belly ends and less around its termination. This is exactly consistent with our findings of site-dependent differences of stiffness within the gastrocnemius aponeurosis (Table 1). On the other hand, The Young’s modulus did not show site-dependent differences in any regions, although there were tendencies in the values being smaller around terminations of both anterior and posterior aponeuroses of the gastrocnemius (Table 2). Thus, the reason for the site-dependent differences in aponeurosis stiffness, is due to different aponeurosis thicknesses rather than a difference in the material property. A previous study reported that the longitudinal stiffness of aponeurosis influences the muscle fascicle behavior and probably favors the magnitude of force production [28], which indicates that the aponeurosis would act to control the muscle fascicle behavior during movement.

### Differences of aponeuroses between gastrocnemii and soleus in morphological and mechanical properties

Compared to other regions of gastrocnemii and soleus, thinner thickness but higher Young’s modulus in the anterior region of GL aponeurosis (longitudinal) and posterior region of SOL-lat aponeurosis (transverse) are somewhat unexpected findings (Figs 4 and 5). Such findings were not found in any other region of GM aponeurosis and SOL-med aponeurosis. The anterior region of GL aponeurosis and posterior region of SOL-lat aponeurosis are adjoining aponeuroses which connect to the Achilles tendon serially. The medial-lateral differences in thickness and Young’s modulus may reflect different muscle-aponeurosis interaction between synergist muscles (GM, GL and soleus), which affects the muscle force transmission to the tendon differently, and contributes differently to the limb movement control between medial and lateral side. A previous cadaveric study on site specificity of structural and mechanical properties of human fascia lata found that higher transverse Young’s modulus accompany with higher proportion of transversely directed fibers [18]. Such differences in aponeuroses morphology and mechanical properties may be related to the unique arrangement of collagenous bundles [29, 30] in the adjoining aponeuroses, which awaits future investigation.

### Direction-dependent differences of mechanical properties

The differences of mechanical properties (stiffness and Young’s modulus) of human triceps surae aponeuroses between longitudinal (along the muscle-tendon unit’s line of action) and transverse (orthogonal to the muscle-tendon unit’s line of action) directions found in the current study are consistent with the features of a turkey’s aponeuroses [16] and human fascia lata [18]. All previous studies indicate that the aponeuroses are more compliant in the transverse direction, and our study further showed that the stiffness and Young’s modulus in the transverse direction were much smaller (<1%) than the values in the longitudinal direction, regardless of regions (Tables 1 and 2). As previously documented [16], most of the collagen fiber bundles of aponeurosis appear to be arrayed longitudinally, so the loading regime of aponeurosis is greatly limited to the longitudinal direction. This may cause the distinct differences between longitudinal and transverse directions in strain. Higher stiffness and Young’s modulus of the aponeurosis in the longitudinal direction, may reflect its role as a mechanical spring within the muscle-tendon unit, whilst being more compliant in the transverse direction possibly helps to accommodate the expansion of the contracting muscle belly in this direction. Compared with the results of turkey’s aponeuroses, the transverse Young’s modulus of human aponeurosis was much lower. One of the possible reasons may be that they used frozen turkey materials while our samples were tested following formalin fixation which may have altered the tissue properties. Another possibility may be the differences in the magnitude of the transverse strain between species (humans: 6–21% [7, 8], turkeys: 8% [6]). On the other hand, another study [18] on human formalin-fixed fascia lata showed similar results of Young’s modulus in the longitudinal direction (71.6–275.9 MPa) as well as in the transverse direction (3.2–41.9 MPa). The anisotropy of the aponeuroses is also far more pronounced in the current study than that reported in a previous *in vivo* human study (Iwanuma et al., 2011). The Young’s modulus determined for the linear region of the stress-strain curve in this study was 100–500 MPa (at 3–5% strain) in the longitudinal direction which was about 100 times higher than in the transverse direction (0.5–3 MPa, at 5–30% strain), whereas an *in vivo* study reported values of the longitudinal strain being only 5–10 times larger than the transverse strain [7]. Their study also found no significant differences in the strains of aponeurosis between 30% maximal voluntary contraction (MVC) and 60% MVC along the longitudinal (1.1 and 1.6%) or transverse (5.0–11.4% and 5.0–13.9%) direction. The elastic feature of aponeurosis in the present study was found in the isolated condition, thus the *in vivo* finding of their study could be due to the condition where contracting muscle fibers were attaching onto the aponeurosis. In this condition, the aponeurosis mechanical properties might be modulated by the stiffened, contracting muscle fibers. The maximal load of aponeurosis during the tensile test of the present study was about 37.8 ± 18.3 N with the strain at 4.9 ± 1.4%. Previous *in vivo* studies reported that the aponeurosis strain was 1.1–1.6% during 30%-60%MVC muscle contractions [7] and 5.9–7% during maximal voluntary contraction [8, 10, 31]. Regarding the aponeurosis strain, the magnitude of elongation in our study is comparable to those under the maximal voluntary contraction *in vivo*. Thus the present findings on inter- and intra-muscle variability in aponeurosis mechanical properties and their anisotropy may relate to the situations of highly active muscle contractions in humans. However, due to substantially variable reported values of aponeurosis strains *in vivo*, and a large number of estimations and assumptions in the parameters to be used for individual muscle forces, we do not feel justified to attempt describing the muscle-aponeurosis behavior during exercises. Future studies to accurately determine individual muscle forces will lead to reasonable and useful modeling of the functional roles of aponeuroses in motor performance.

### Viscoelasticity of triceps surae aponeuroses

The aponeurosis serially connects to tendon which enables it to transmit the forces produced from muscle fibers to tendon, and finally to the bones. However, the work produced by muscle fibers cannot be fully transmitted to the bone due to the viscoelasticity of aponeurosis and tendon tissues. The energy dissipation during loading and unloading process is called hysteresis, and tendon hysteresis has been measured in *in vivo* human studies [32–35] with diverse values between studies. A previous study suggested that hysteresis is a quite sensitive measure largely influenced by the method utilized [36]. In our results, the energy dissipation along longitudinal and transverse directions was different, with the former being smaller than the latter (Table 3). The aponeuroses were much softer transversely, and during the unloading phase, more energy was lost (dissipated as heat), so the efficiency (unloading energy/ loading energy) decreased. Since the amount of hysteresis may influence the efficiency of muscle contraction with the same conformation [35, 37, 38], lower hysteresis in the longitudinal direction may make the muscle more efficient, which can relate to the high efficiency of human stretch-shortening cycle movement such as walking where the triceps surae are the major agonists [39].

### Implications for muscle-tendon-aponeuroses interaction during contraction

A previous study stated that the elastic mechanism (e.g. energy conservation vs. power amplification) of biological springy tissues (such as tendinous tissue) is important for the effective function of muscle-tendon complex to enhance movement [40], since such a mechanism will allow the locomotor system to operate by not only the muscle motors but by the interactions between muscle motors and elastic tendon springs. A previous study [6] showed the variation in the mechanical properties of aponeurosis with biaxial loading during active force production, and another *in vivo* study [7] found no significant changes of aponeurosis strains in the longitudinal direction between 30% and 60% MVC, while the free tendon was deformed. As described earlier, the elasticity of the aponeurosis, and hence the use of elastic energy thereof, may depend on the muscle contraction levels. The mechanical properties of the aponeurosis could allow for the muscle belly deformation at low force levels, while limiting muscle fibers’ further deviation longitudinally at higher force levels so that the free tendon can behave like a spring during movements. Future work on *in vivo* study should consider how the inter-muscle and inter-direction differences of triceps surae aponeuroses elasticity depend on muscle contraction levels.

### Limitations

The limitations of our study should be noted. Firstly, all the specimens were dissected from formalin-fixed cadavers. Previous studies [41, 42] found that formalin fixation decreased stiffness and Young’s modulus of human femur-ACL-tibial complex significantly compared to the fresh tissues (as for stiffness, fresh: 166.45 ± 36.03 N/mm, formalin-fixed: 71.68 ± 5.05 N/mm; as for Young’s modulus, fresh: 71.5 ± 18.67 N/mm, formalin-fixed: 31.77 ± 2.52 N/mm) and formalin-fixed bones showed a significantly lower Young’s modulus (-12%) compared to the fresh tissues. On the other hand, the cadavers in the current study were donated from elderly individuals. An existing study [43] proved that the donor age can affect mechanical properties of human Achilles tendon, and many studies [44–46] have found higher tendinous compliance in elderly individuals. Therefore, the conclusion of the present study may be limited to the characteristics of the muscle-tendon unit in the elderly. Anisotropic mechanical behavior of the soft tissues may also be affected by fixation although there has been no such report to date. Therefore, we conducted additional data collection to examine the effect of formalin on the mechanical properties of aponeuroses by using urea that has been reported to neutralize formaldehyde within cadaveric tissues without affecting cadaveric and histological quality [47]. The results (S2 Fig) showed that the Young’s modulus was slightly but significantly increased (pre: 143.1 ± 77.3 MPa, post: 157.3 ± 79.8 MPa) only in the longitudinal direction after the urea treatment, while there was no significant change for the transverse direction or longitudinal/transverse ratio (Table A in S1 File). This suggests that the anisotropic mechanical behavior would already have existed before formalin fixation. Additionally, we used 50% alcohol (an agent that dehydrates tissue) instead of normal saline solution to keep the specimens, and to check whether this dehydrating agent potentially impacts our results or not, an additional experiment was carried out. The results (S1 Fig and S2 Fig) showed that the moisture content of triceps surae aponeuroses was not changed significantly, and there were no significant changes in the Young’s modulus either in the longitudinal or transverse direction after 50% alcohol treatment for 5 hours. Although we tested each specimen twice in two directions with a randomized counterbalanced order, it may still have influenced the properties between longitudinal and transverse directions. Our additional experiments with one specimen for only one direction showed similar results (S2 Fig), but this can be taken into consideration for the future studies. The present results will open the possibility of understanding how aponeuroses mechanical properties are related with the muscle-aponeurosis-tendon behavior and/or interactions between the gastrocnemii and soleus during contractions *in vivo*.

## Conclusions

In the present study, site-related differences of thickness were found from proximal to distal in triceps surae aponeuroses, with different morphology and mechanical properties among aponeuroses of synergist muscles. The reason for site-dependent differences in stiffness is due to a reduced aponeurosis thickness rather than a reduction in the material property. The anisotropic elastic feature (differences between longitudinal and transverse directions in stiffness and Young’s modulus) of the aponeuroses were more pronounced than *in vivo* observations, suggesting inherent material design of the aponeurosis that matches three-dimensional contractile behavior of muscle fibers.

## Supporting information

S1 File Methods and results of moisture content test and additional tensile test. (DOCX)

S1 Fig Moisture content test on aponeuroses samples from a cadaver. (A) A moisture analyzer with a testing specimen. (B) Average (mean ± s.d.) moisture content of triceps surae aponeuroses before placing any solution (Pre), after normal saline solution for 5hr and after 50% alcohol solution for 5hr.
(TIF)

S2 Fig Mechanical properties of triceps surae aponeuroses with different solution method. Average (mean ± s.d.) Young’s modulus of triceps surae aponeuroses in the longitudinal (A) and transverse (B) directions before (Pre) and after (Post) normal saline, 50% alcohol and 18% urea treatment. *: denotes different from pre, *p* < 0.05.
(TIF)
