# Supplementary material for: Morphological and mechanical properties of the human triceps surae aponeuroses taken from elderly cadavers: Implications for muscle-tendon interactions
Source: PLoS One. 2019 Feb 8;14(2):e0211485. doi: 10.1371/journal.pone.0211485 (PMC6368299; doi:10.1371/journal.pone.0211485)
Supplement: S1 File — (DOCX) [file pone.0211485.s001.docx]

**Materials and methods**

**Moisture content test**

Eight specimens were taken out from various regions of the triceps surae aponeuroses of one cadaver (formalin fixed, 83 years, male). Next, each specimen was divided into 3 parts, so the specimens were assigned to 3 groups, which were further kept in 20% formaldehyde (Pre), before being kept in the saline solution for 5 hours, or before being kept in the 50% alcohol for 5 hours. A ML-50 moisture analyzer (A&D Co., Ltd., Tokyo, Japan) was used to measure the moisture content with the standard mode.

**Tensile test**

Six pairs of triceps surae muscles were dissected from three cadavers (formalin fixed, 90-95 years, female). Each two pairs were assigned either to a saline or alcohol or urea group, and specimens of aponeuroses were dissected from individual muscles (saline group: longitudinal, n = 10; transverse, n = 9; alcohol group: longitudinal, n = 8; transverse, n = 8; urea group: longitudinal, n = 10; transverse, n = 8). Each specimen was applied to tensile test only in one direction (longitudinal or transverse). The uniaxial tensile test was implemented before (Pre) and after (Post) normal saline solution (5 hr), 50% alcohol (5 hr) and 18% urea solution (5 hr). The strain rate of tensile test for each specimen was kept identical between Pre and Post, and stiffness and Young’s modulus were calculated from the linear region of the force-displacement relationship and stress-strain relationship curve, respectively.

**Statistics**

A paired t-test was used to examine the moisture content changes after the normal saline and 50% alcohol treatments. A paired t-test was used to examine the stiffness and Young’s modulus changes after the normal saline, 50% alcohol and 18% urea treatments, both in the longitudinal and transverse directions. A paired t-test was used to examine the changes of longitudinal/transverse ratio after 18% urea treatment. The significance level was set at *α* < 0.05.

**Results**

There were no significant changes of moisture content both after normal saline solution (Pre vs. Post, 64.5 ± 4.2% vs. 62.1 ± 5.6%) and 50% alcohol (Pre vs. Post, 64.5 ± 4.2% vs. 62.8 ± 5.9%) (S1 Fig). There were no significant changes of Young’s modulus both in the longitudinal and transverse directions after normal saline solution (longitudinal: Pre vs. Post, 106.6 ± 67.2 MPa vs. 105.9 ± 66.4 MPa; transverse: Pre vs. Post, 2.6 ± 2.8 MPa vs. 2.3 ± 2.4 MPa) and 50% alcohol solution (longitudinal: Pre vs. Post, 136.0 ± 54.0 MPa vs. 126.8 ± 45.6 MPa; transverse: Pre vs. Post, 1.5 ± 0.7 MPa vs. 1.6 ± 0.5 MPa) (S2 Fig). The Young’s modulus was slightly but significantly increased (pre: 143.1 ± 77.3 MPa, post: 157.3 ± 79.8 MPa) only in the longitudinal direction after the urea treatment, while there was no significant change for the transverse direction or longitudinal/transverse ratio (Table A).

**Table A. Longitudinal/transverse ratio of Young's modulus before and after 18% urea treatment**

|  | **Pre** |  | **Post** |  |  |
| --- | --- | --- | --- | --- | --- |
| **Urea** | 111.7 ± 54.1 |  | 113.2 ± 65.4 |  |  |
