# Supplementary figures and images for: Morphological and mechanical properties of the human triceps surae aponeuroses taken from elderly cadavers: Implications for muscle-tendon interactions
Source: PLoS One. 2019 Feb 8;14(2):e0211485. doi: 10.1371/journal.pone.0211485 (PMC6368299; doi:10.1371/journal.pone.0211485)

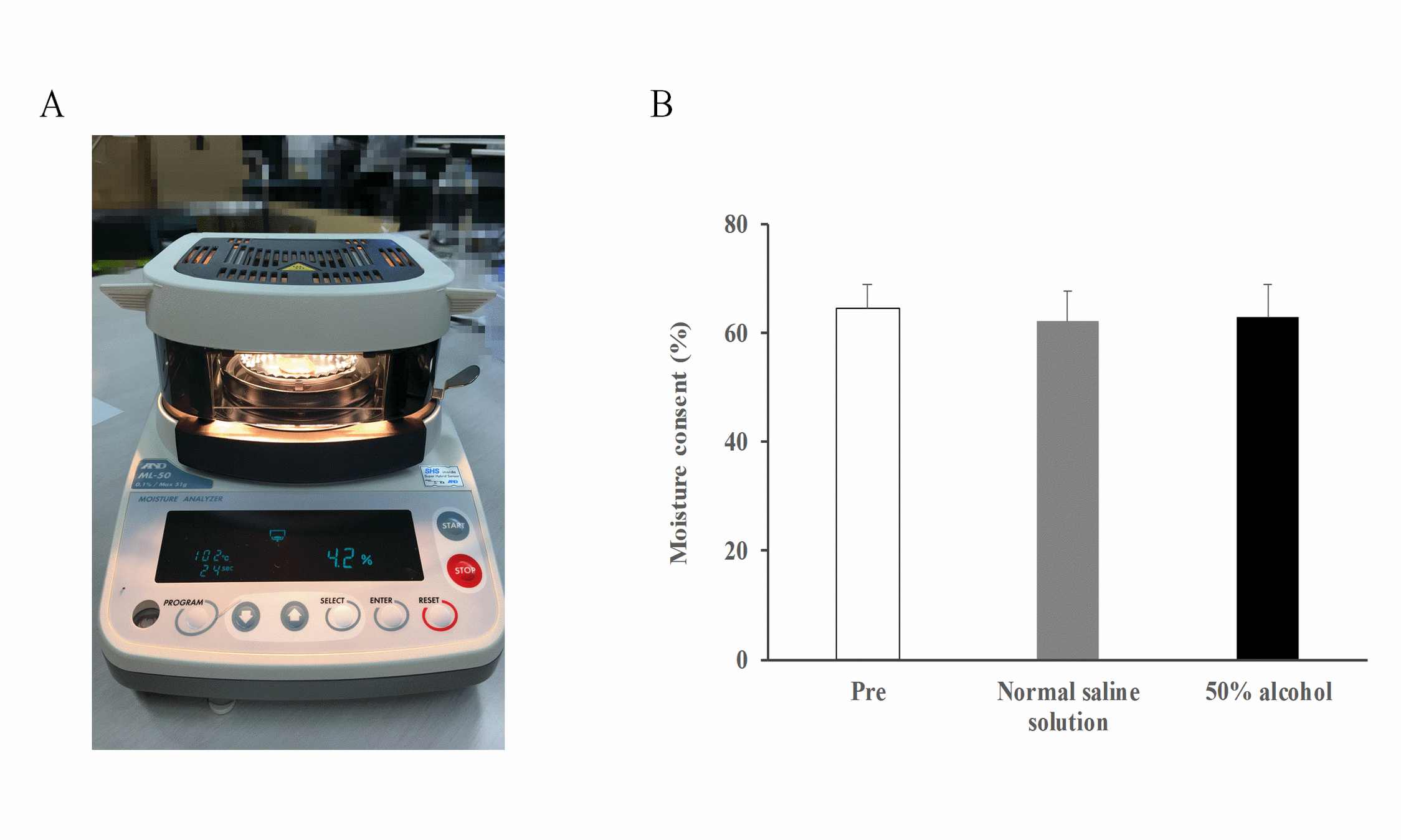

Supplement: S1 Fig — (A) A moisture analyzer with a testing specimen. (B) Average (mean ± s.d.) moisture content of triceps surae aponeuroses before placing any solution (Pre), after normal saline solution for 5hr and after 50% alcohol solution for 5hr. (TIF) [file pone.0211485.s002.tif]

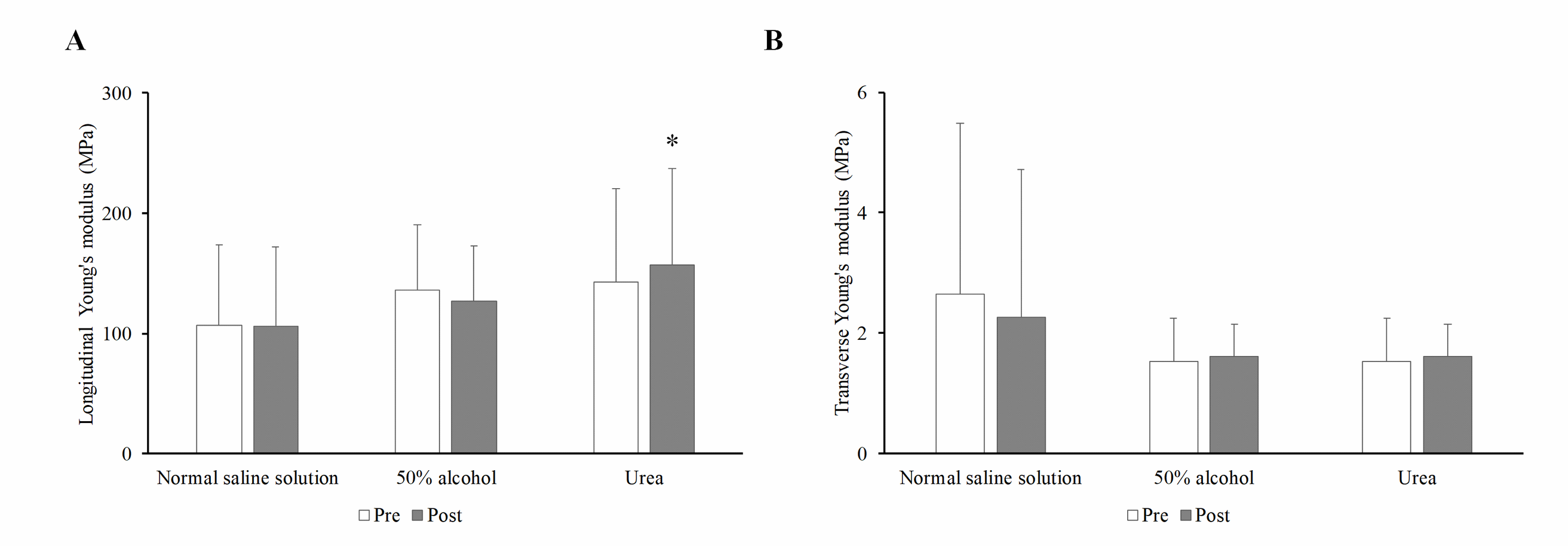

Supplement: S2 Fig — Average (mean ± s.d.) Young’s modulus of triceps surae aponeuroses in the longitudinal (A) and transverse (B) directions before (Pre) and after (Post) normal saline, 50% alcohol and 18% urea treatment. *: denotes different from pre, p < 0.05. (TIF) [file pone.0211485.s003.tif]
